# Alteration of Structure and Characteristics of Concrete with Coconut Shell as a Substitution of a Part of Coarse Aggregate

**DOI:** 10.3390/ma16124422

**Published:** 2023-06-15

**Authors:** Sergey A. Stel’makh, Alexey N. Beskopylny, Evgenii M. Shcherban’, Levon R. Mailyan, Besarion Meskhi, Alexandr A. Shilov, Diana El’shaeva, Andrei Chernil’nik, Svetlana Kurilova

**Affiliations:** 1 Department of Unique Buildings and Constructions Engineering, Don State Technical University, 344003 Rostov-on-Don, Russia; lrm@aaanet.ru (L.R.M.); alexandr_shilov@inbox.ru (A.A.S.); diana.elshaeva@yandex.ru (D.E.); chernila_a@mail.ru (A.C.); 2 Department of Transport Systems, Faculty of Roads and Transport Systems, Don State Technical University, 344003 Rostov-on-Don, Russia; 3 Department of Engineering Geology, Bases and Foundations, Don State Technical University, 344003 Rostov-on-Don, Russia; au-geen@mail.ru; 4 Department of Life Safety and Environmental Protection, Faculty of Life Safety and Environmental Engineering, Don State Technical University, 344003 Rostov-on-Don, Russia; spu-02@donstu.ru; 5 Department of Building Materials, Don State Technical University, 344003 Rostov-on-Don, Russia; svet.curilova@yandex.ru

**Keywords:** concrete, sustainable concrete, coconut shell, natural coarse aggregate, compressive strength

## Abstract

One of the most promising ways to solve the problem of reducing the rate of depletion of natural non-renewable components of concrete is their complete or partial replacement with renewable plant counterparts that are industrial and agricultural waste. The research significance of this article lies in the determination at the micro- and macro-levels of the principles of the relationship between the composition, the process of structure formation and the formation of properties of concrete based on coconut shells (CSs), as well as the substantiation at the micro- and macro-levels of the effectiveness of such a solution from the point of view of fundamental and applied materials science. The aim of this study was to solve the problem of substantiating the feasibility of concrete consisting of a mineral cement–sand matrix and aggregate in the form of crushed CS, as well as finding a rational combination of components and studying the structure and characteristics of concrete. Test samples were manufactured with a partial substitution of natural coarse aggregate with CS in an amount from 0% to 30% in increments of 5% by volume. The following main characteristics have been studied: density, compressive strength, bending strength and prism strength. The study used regulatory testing and scanning electron microscopy. The density of concrete decreased to 9.1% with increasing the CS content to 30%. The highest values for the strength characteristics and coefficient of construction quality (CCQ) were recorded for concretes containing 5% CS: compressive strength—38.0 MPa, prism strength—28.9 MPa, bending strength—6.1 MPa and CCQ—0.01731 MPa × m^3^/kg. The increase in compressive strength was 4.1%, prismatic strength—4.0%, bending strength—3.4% and CCQ—6.1% compared with concrete without CS. Increasing the CS content from 10% to 30% inevitably led to a significant drop in the strength characteristics (up to 42%) compared with concrete without CS. Analysis of the microstructure of concrete containing CS instead of part of the natural coarse aggregate revealed that the cement paste penetrates into the pores of the CS, thereby creating good adhesion of this aggregate to the cement–sand matrix.

## 1. Introduction

The relevance of the ongoing research is due to the environmental agenda and the economic feasibility of finding new recipes and technologies for building materials in general, and concrete in particular, that can be used in sustainable construction. The concept of sustainable development implies a full cycle of waste disposal that occurs in various spheres of human activity, enterprise and society [1,2,3]. Of course, growing fruits and crops are important aspects of the economies of many countries. These countries, in their economic development, reflect the need for the development of agriculture but at the same time all plant production is characterized by an accompanying problem. This problem is the accumulation of waste of plant origin in the absence or shortage of rational methods for their disposal [4,5]. This is why modern scientists direct applied as well as fundamental research to determine the fundamental nature of and, thereby, substantiate the practical expediency of the applicability of plant waste in other areas of the economy. One of the most suitable areas for the disposal of plant waste is in building materials science and the production of building materials [6,7,8,9,10,11,12]. At the same time, materials scientists are faced with the question of studying the fundamental nature of the processes and physicochemical processes that take place during the formation of the structure and properties of building materials using plant waste. Of particular difficulty and at the same level of interest is the determination of the most rational quantities and the degree of compatibility and effectiveness of the combination of plant wastes in the body of mineral components of other materials, including concrete [5,13,14,15]. Recently, the question of the inconsistency of the concept of sustainable development with this approach of using natural mineral resources, for example river sand and crushed stone, for the needs of the construction industry has been increasingly raised [16]. It is becoming promising to develop technologies that can reduce the rate of depletion of natural non-renewable components of concrete [4,17,18]. One of the most promising methods to solve this problem is the complete or partial substitution of the mineral components of concrete with natural renewable plant analogues that are industrial and agricultural waste; this solves a number of problems for these industries [14,15,19,20]. However, new renewable materials should not reduce the strength characteristics of concrete or adversely affect its durability [21].

**Figure 1:**
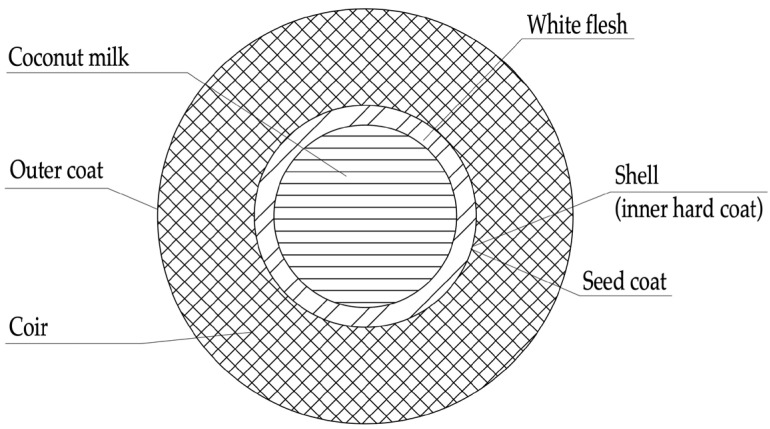
The structure of the fruit of the coconut palm.

The promising candidate for partial replacement of the mineral components of concrete is machined coconut shell (CS), which has good potential to become an alternative to coarse aggregate in concrete [22,23,24,25]. CS is a by-product of the agricultural processing of coconuts. It is part of the endocarp of the coconut, namely a strong shell that protects the seed from mechanical stress. A schematic composition of the coconut palm fruit is shown in Figure 1.

**Figure 2:**
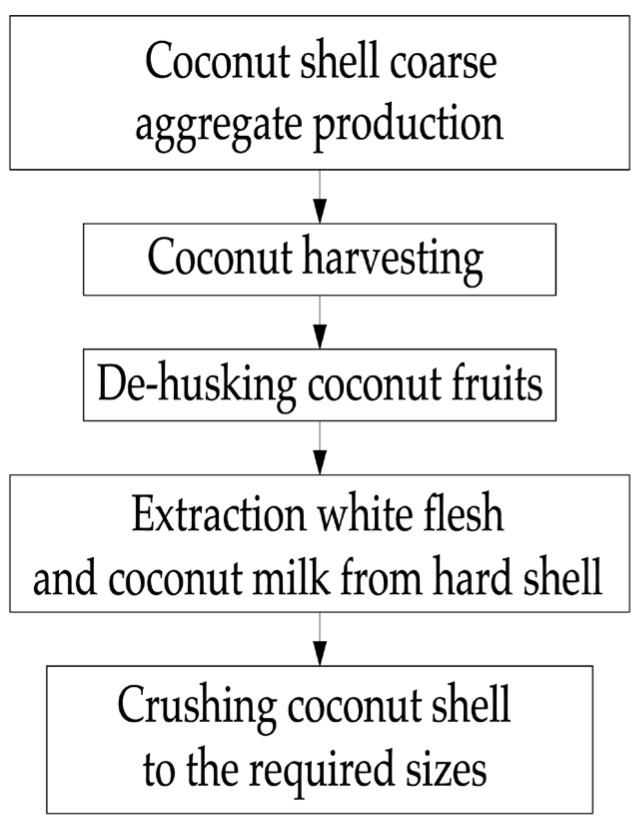
Manufacturing process of coarse aggregate for concrete from CS.

After harvesting the fruit of the coconut palm and separating the fibrous part (coir) from the coconut, the CS is mechanically opened and discarded after the pulp has been removed. Both the burial and incineration of the CS waste are often used; both methods, as mentioned earlier, are harmful to the environment [5,26]. The use of CS as a partial or complete replacement for large aggregates in concrete will make it possible to utilize large volumes of this raw material practically without waste or significant damage to the environment [27,28]. The process of making coarse aggregate for concrete from CS is shown in Figure 2.

Therefore, CS is a natural raw material with a high degree of renewal, which makes it possible to use it in significant volumes without the risk of depleting it. Additionally, the application of CS in the composition or reinforcement of materials is presented in a number of the following works [13,29,30,31,32,33,34,35,36,37,38,39,40,41,42,43,44,45,46,47,48]. The introduction of CS as a component is studied in detail in [29,30]. It was shown in [29] that using CS as 53% of the fine aggregate was the most optimal amount and made it possible to obtain concrete with a lower thermal conductivity (0.59 W/m K) than concrete with the control composition (0.76 W/m TO). The study in [30] evaluated the permeability of concrete with the introduction of CS as a fine component. It was established that replacement of up to 50% of the fine aggregate with CS is rational and makes it possible to obtain good quality concretes with low penetrating quality (less than 2.7 × 10^−11^ m/s) and water absorption up to 5%. In the studies [31,32,33,34,35,45], the authors studied the possibility of using CS as a coarse aggregate in the technology of heavy concrete. For example, in [31], the authors found that the use of CS in concrete as a coarse aggregate in an amount of 10–20% “has a negative effect on the strength characteristics of concrete” and contributes to an increase in drying shrinkage. In one study [32], the authors estimated the strength properties of concrete with partial replacement of coarse aggregate with CS. It was found that CS concretes have a moderate permeability to chloride ions. In the works [33,34,35,45], the authors reported that the inclusion of CS in heavy concrete in rational quantities made it possible to produce concrete without deteriorating performance. In [13,36,37,38,39,40], lightweight concretes with the addition of CS were studied. In study [36], it was found that with an increase in the CS content, the density and strength of lightweight structural concrete decreased. In [37], the authors studied the properties of high-strength lightweight concretes with crushed CS as a coarse component and found that the use of this aggregate in rational amounts made it possible to obtain high-strength lightweight concretes with characteristics in terms of compressive strength, elastic modulus and tensile strength in splitting that were approximately equal to the concretes of the control composition. In [38], lightweight self-compacting concretes with 75% CS content showed good results in properties such as water absorption, sorption capacity and resistance to chloride penetration, which were comparable to the results for concretes of the control composition. The results obtained in studies [13,39] prove the possibility of using CS waste as an aggregate in the technology of lightweight structural concrete without significant deterioration in the properties of these concretes at rationally selected amounts of the CS waste and the other concrete components.

In [41,42,43,44,45,46,47], the behavior and properties of various reinforced concrete elements made using CS were studied. For example, in [41], the authors produced and studied two series of reinforced plates for manhole covers that were 600 × 600 × 100 mm in size. One series of covers was made on normal concrete and the second series was made on concrete with CS. Additionally, steel fibers and microsilica were added to the concrete mix. The test results showed that the performance characteristics of concrete caps with coconut filler were approximately comparable with those of conventional concrete caps, meeting the regulatory requirements and being suitable for use. In [42], the authors proved the possibility of using concrete beams containing a 5% replacement of coarse CS and a 10% replacement of part of the cement with coconut ash as an alternative to traditional reinforced concrete beams. In [43], the characteristics of plastic shrinkage and the deflection of concrete slabs with different CS contents were studied. It was found that in slabs with CS there was a decrease in plastic shrinkage cracking; however, at the same time there were deformability increases compared with slabs made of ordinary concrete.

Summarizing the literature and analyzing the references devoted to research on the use of coconut shell in concrete, specific research scientific problems can be identified. One of the main problems is the lack of clearly formulated mechanisms and explanations of the physical and chemical processes that occur during the formation of the structure of an organo-mineral composite, including a cement–sand matrix and a coconut shell aggregate, from the fundamental and applied point of view. Such a generalizing experience will be extremely useful for forming a new scientific and technical basis for future research, as well as for applied developments carried out by engineers and concrete factories, especially in interested countries with a large amount of coconut waste.

The above brief review shows that the use of coconut shell as a replacement for part of the coarse filler is an interesting and relevant scientific problem. However, there is a gap in this area. The processes of the formation of the structure of concrete containing coconut shells has not been studied enough. The strength characteristics of concrete and the optimal compositions of the concrete mix have not been quantitatively evaluated. Thus, the scientific novelty of the study lies in the establishment of new dependencies and relationships of a fundamental and applied nature that occur during the formation of the structure and properties of an artificial organo-mineral conglomerate—a coconut shell in the body of a cement concrete matrix. These new dependencies and relationships are the chains we have obtained:

− “the composition of the conglomerate—the microstructure of the boundaries of the phases of the conglomerate”. The macrostructure of the conglomerate—the properties of concrete. The applied methodological and phenomenological approaches made it possible to provide a high degree of verification of the results. These approaches are primarily characterized by the observance of the fundamental principle of materials science “composition—structure—properties”, as well as the determination of the most characteristic, relative indicator for non-standard concretes—the coefficient of constructive quality.

In this study, the scientific hypothesis is the possibility of obtaining high-quality building concrete consisting of a mineral cement–sand matrix and aggregate in the form of plant waste, namely crushed CS. The aim of the study is to solve the problem of substantiating the feasibility of such concrete, as well as to find a rational combination of components and study the structure and characteristics of concrete with CS as a replacement for coarse aggregate. Thus, two research problems are solved. Fundamental—study of the mechanisms of structure formation and the formation of properties of concrete using CS. Applied—searching for a rational combination of components and identifying the influence of various recipes and technological factors on the structure and properties of concretes based on CS and, thereby, finding the optimal value of the ratio of concrete strength to its density when using the maximum possible amount of plant waste (in the form of CS) as an aggregate in concrete.

The practical significance of the study thus lies in obtaining an important performance indicator of the proposed concrete, which can be used in the calculations and design of lightweight coco-concrete eco-structures.

## 2. Materials and Methods

### 2.1. Materials

Portland cement CEM I 42.5N (CEMROS, Stary Oskol, Russia), which does not contain additives, was used as a binder for the manufacture of prototypes. The main characteristics and mineralogical composition of the Portland cement used in the study are presented in Table 1 and Table 2.

Table 2 data provided by cement manufacturer. Fine aggregate is represented by sand (LLC “DON-RESURS”, Kagalnik, Russia). The characteristics of the fine aggregate are shown in Table 3.

Crushed sandstone (RostMed, Kamensk, Russia) and CS (Auriki Gardens, Yaroslavl, Russia) were used as natural coarse aggregates. The shell was stored in a dry, ventilated room at an air temperature of 25 °C and a humidity of 65% for at least 48 h before use. The characteristics of crushed sandstone are presented in Table 4 and those of CS in Table 5.

**Figure 3:**
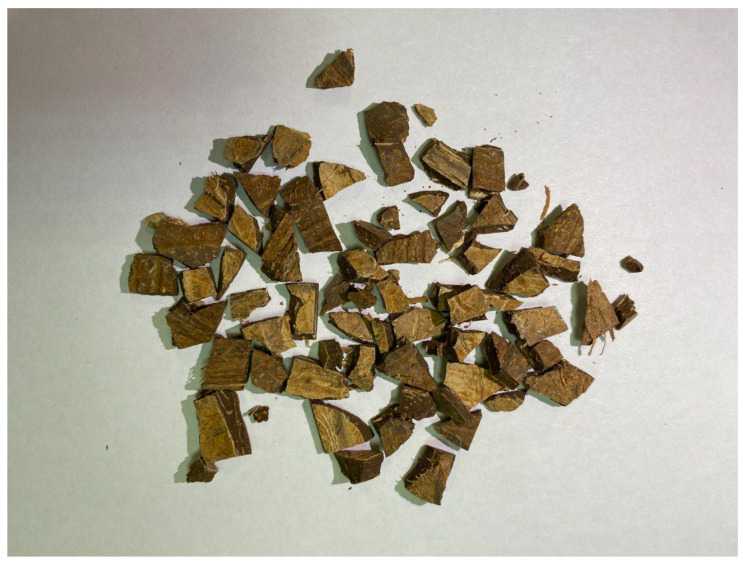
Appearance of the applied CS.

A general view of the CS used to make the samples is shown in Figure 3.

The superplasticizer Poliplast-SP1 (LLC Poliplast-South, Krasnodar, Russia) was used as a plasticizing additive. This additive is a dark brown aqueous solution with a density of 1.17 g/cm^3^ and pH 8 ± 1.

### 2.2. Methods

The designs of the concrete mixture of the control composition and the mixtures with different CS contents are compiled in Table 6.

The choice of the proportions of the concrete mixtures (water–cement ratio, percentage of CS and other parameters) was based on the concrete compositions already selected by us in previous works [14,15] and the works of other authors [33,34,46], with their adjustment related to the properties of the components used [51].

With the partial replacement of natural coarse aggregate with coconut shell, the water demand of the concrete mixture increases due to the greater porosity of the structure of the organic component [52]. Accordingly, the slump of the mixture decreases with increasing coconut shell content. Therefore, to maintain the same workability for all mixtures, a superplasticizer was used in the study.

**Figure 4:**
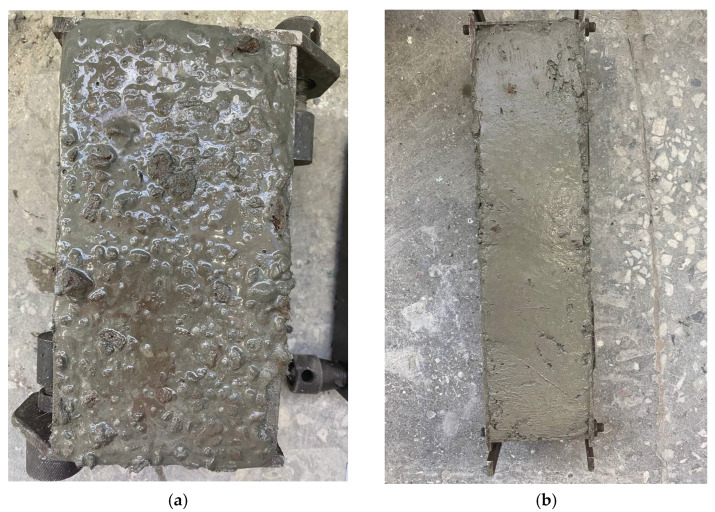
Freshly made concrete samples: (**a**) cubes; (**b**) prisms.

Freshly made sample cubes and prisms of concrete are shown in Figure 4.

The manufacture of concrete mixtures and laboratory samples was developed in the following sequence: measurement of the quantities of the raw materials and preliminary mixing of these components in dry mold; the inclusion of mixing water with a plasticizing additive; mixing the mixture until homogeneous; laying the mixture in molds; vibrating molds for 60 s. The finished samples were kept for 1 day and removed from the molds. Then, these samples were kept for 27 days in a normal hardening chamber at a temperature of 20 ± 2 °C and a relative air humidity of 95% [53].

For the manufacture of concrete mixtures and concrete samples, the following technological equipment was used:

− Laboratory concrete mixer BL-10 (ZZBO, Zlatoust, Russia);
− Laboratory scales HT-5000 (NPP Gosmetr, St. Petersburg, Russia);
− Cube shape 2FK-100 and beam shape FB-400 (RNPO RusPribor, St. Petersburg, Russia);
− Normal hardening chamber KNT-1 (RNPO RusPribor, St. Petersburg, Russia).

**Figure 5:**
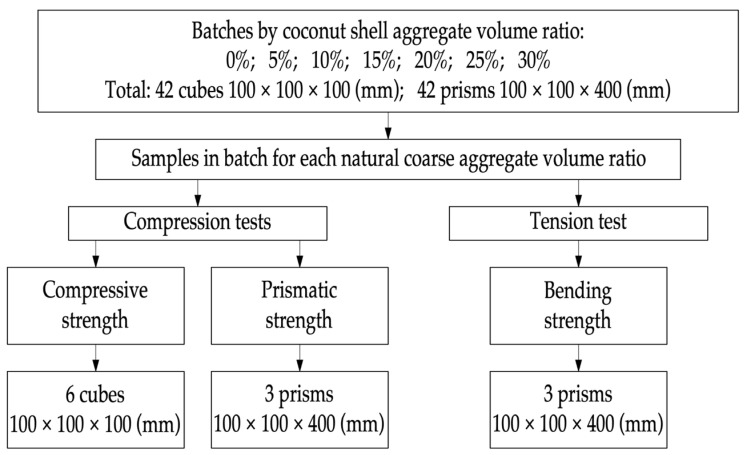
Mechanical test program.

The program of experimental research is shown in Figure 5.

The assessments of the compressive strength and bending strength (four-point) were produced in accordance with document [53], the requirements of which correspond to the main regulations regarding the manufacture and testing of concrete samples given in the following European regional standards [54,55,56,57,58].

Prism strength was determined in accordance with document [59], corresponding to the main regulations given in ASTM C1314-23 “Standard Test Method for Compressive Strength of Masonry Prisms”.

The compressive strength was calculated by Formula (1):

(1) $$R=\alpha \frac{F}{A}$$

where *F* is the breaking load (N), *A* is the area of the working section of the sample (mm) and α is the scale factor (for cubes with a rib size of 100 mm it is equal to 0.95).

**Figure 6:**
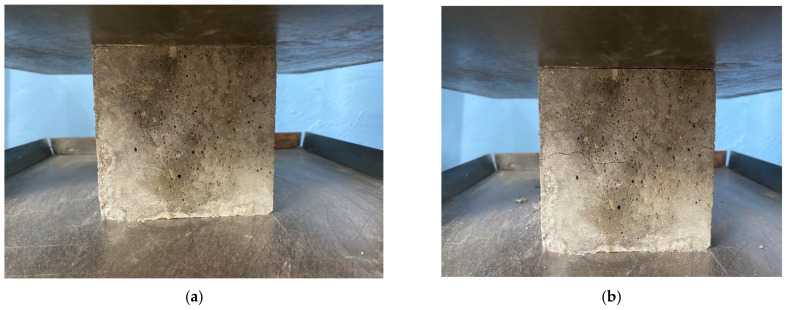
Compressive strength testing of concrete specimens: (**a**) specimen before failure; (**b**) sample at the time of destruction.

**Figure 7:**
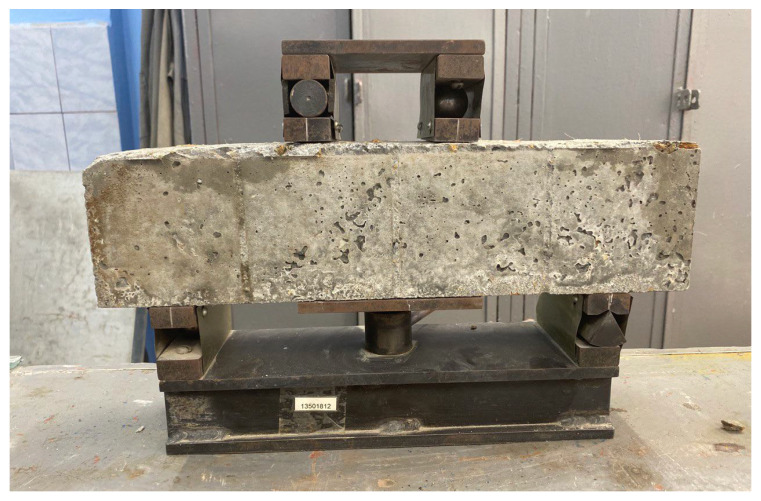
Four-point bend test setup.

**Figure 8:**
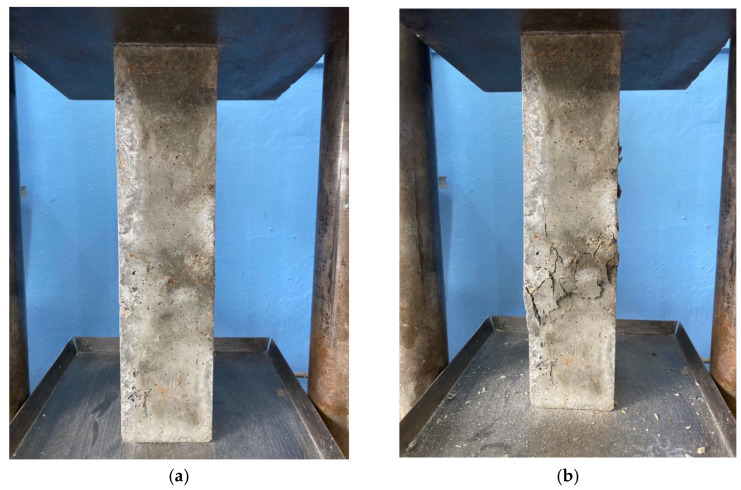
The process of testing concrete specimens for prismatic strength: (**a**) sample before failure; (**b**) sample at the time of destruction.

The process of determining the compressive strength, bending strength and prismatic strength of the samples is presented in Figure 6, Figure 7 and Figure 8, respectively.

The bending strength was calculated by Formula (2):

(2) $$R_{tb}=\delta \frac{Fl}{ab^{2}}$$

where *l* is the distance between the supports (mm), *a* and *b* are the width and height of the cross section of the prism, respectively, and *δ* is the scale factor (for cubes with a rib size of 100 mm it is equal to 0.92).

Prism strength was calculated by Formula (3):

(3) $$R_{pr}=\frac{F}{A}$$

The density of hardened concrete was determined according to [60]; the main provisions of which are in accordance with EN 12390-7:2019 “Testing hardened concrete—Part 7: Density of hardened concrete”.

The density was calculated using Formula (4):

(4) $$\rho_{w}=1000\frac{m}{V}$$

where *m* is the mass of the sample (g) and *V* is the sample volume (cm^3^).

The coefficient of constructive quality (*CCQ_R_*) determined by compressive strength *R* was calculated by Formula (5):

(5) $$CCQ_{R}=\frac{R}{\rho }$$

where *R* is compressive strength (MPa) and *ρ* is the density of hardened concrete (g/cm^3^) [61,62].

## 3. Results and Discussion

### 3.1. Study of the Physical and Mechanical Characteristics of Concrete

**Figure 9:**
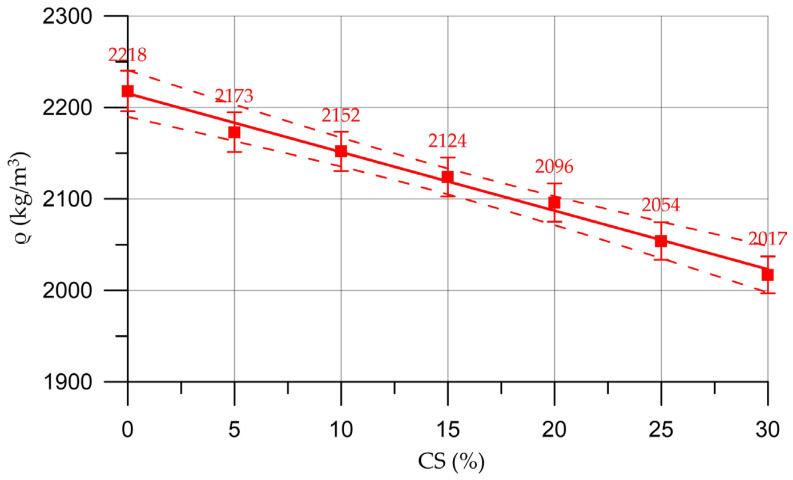
Relationship of concrete density with the proportion of CS.

**Figure 10:**
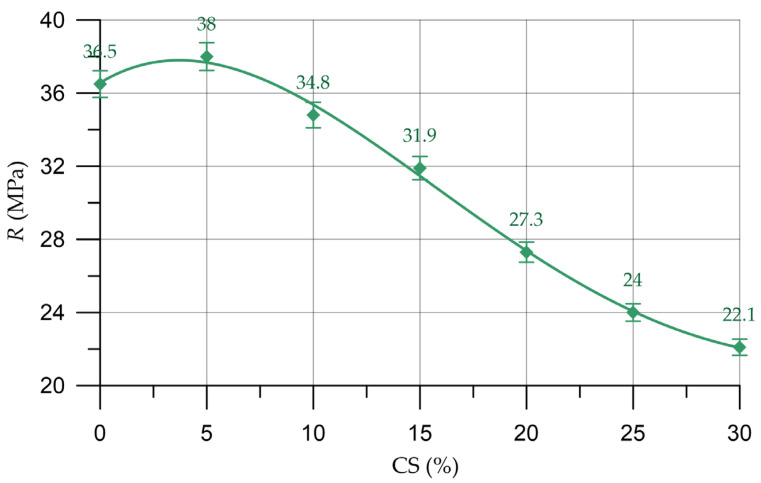
Relationship of concrete compressive strength with the proportion of CS.

**Figure 11:**
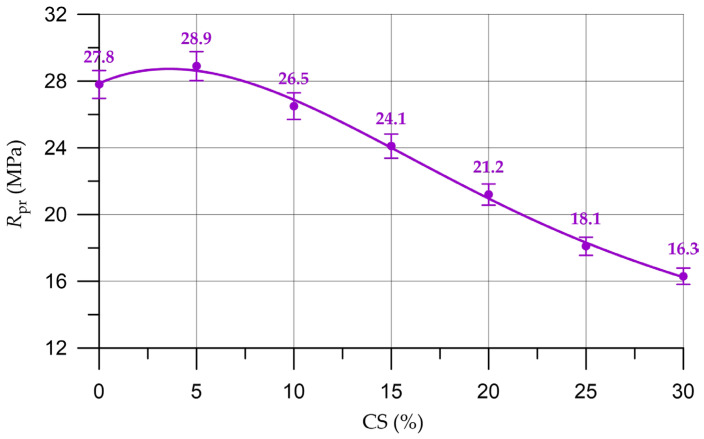
Relationship of the prism strength of concrete with the proportion of CS.

**Figure 12:**
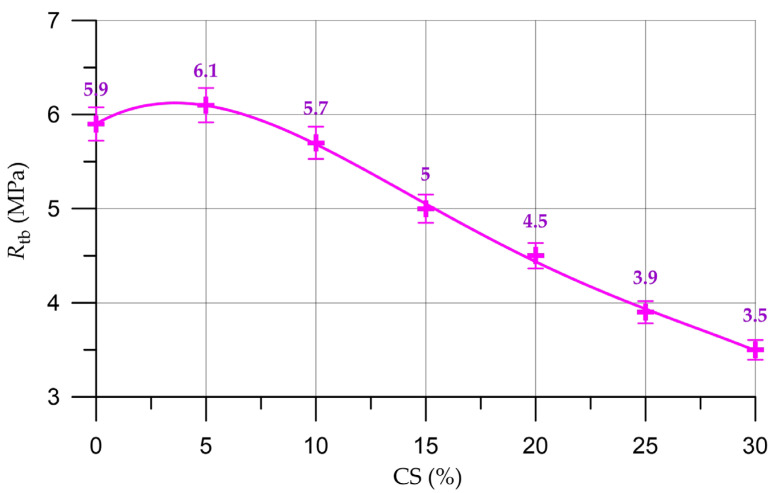
Relationship of concrete flexural strength with the proportion of CS.

The effects of the partial replacement of natural coarse aggregate with organic CS aggregate on concrete density (ρ), compressive strength (*R*), prismatic strength (*R*_pr_) and flexural strength (*R*_tb_) are shown in Figure 9, Figure 10, Figure 11 and Figure 12, respectively.

Alterations in the characteristics of concrete depending on the proportion of CS introduced to substitute a part of the natural coarse aggregate are shown in Table 7 and are presented as a percentage compared with the control composition.

The dependencies shown in Figure 9, Figure 10, Figure 11 and Figure 12 are approximated by polynomial Functions (6)–(9) of the following form:

(6) $$\rho =-6.407\;x+2215,\;R_{d}^{2}=0.991$$

(7) $$R=36.570+0.7053\;x\;-0.112\;x^{2}+0.00328\;x^{3}-2.848\times 10^{-5}\;x^{4},\;R_{d}^{2}=0.997$$

(8) $$R_{pr}=27.87+0.5125\;x\;-0.0846\;x^{2}+0.00262\;x^{3}-2.667\times 10^{-5}\;x^{4},\;R_{d}^{2}=0.997$$

(9) $$R_{tb}=5.902+0.1324\;x\;-0.02248\;x^{2}+0.000804\;x^{3}-9.697\times 10^{-6}\;x^{4},\;R_{d}^{2}=0.998$$

Equations (6)–(9) show the high level of the determination coefficient $R_{d}^{2}$. The evaluation of the coefficients of the regression equations in the form of Equations (6)–(10) was carried out using the least squares method. The assessment of the significance of the regression equation as a whole was carried out using the Fisher test with the F-criterion at a significance level of α = 0.05. In this case, the null hypothesis (H_0_) is put forward: the regression coefficient is zero (b = 0); therefore, the factor x does not affect the result y and the regression line is parallel to the x-axis. For all equations, the regression coefficients are significant with a high degree of confidence.

From Figure 9 and Table 7, it can be seen that with an increase in the percentage of CS as a replacement for coarse aggregate, the density of the concrete decreases. The dependence of the change in concrete density on the percentage of CS content is almost linear.

By analyzing the results presented in Figure 10, Figure 11 and Figure 12 and Table 7, it was established that the highest compressive strength, prismatic strength and bending strength were determined for composition of concrete with a CS content of 5% (which was used instead of a part of the natural coarse aggregate). In comparison with the control composition, the enlargement in the compressive strength was 4.1%, the prismatic strength grew by 4.0% and the bending strength increased by 3.4%. This slight increase in strength properties takes place on the basis of the natural properties of CS. Due to its rough and porous surface, the CS introduced as a replacement for part of the natural aggregate has a fairly high degree of adhesion to the mortar part and acts as an additional sealing component in the cement–sand mortar coarse aggregate. During the production of the concrete mixture, the CS absorbs a small amount of water; then, in the process of the hardening of the cement composite, this water is used as a reserve and the hydration process is more intensive at the phase boundaries [52]. Almost all cement particles enter into a hydration reaction and additional crystallization centers are formed, which, in turn, increase the adhesion strength of CS with the components of the concrete mixture. This is also in good agreement with other studies [33,34,52,63,64]. However, this effect is not so significant, and further increases in the CS content of concrete have no effect and the strength characteristics decrease, which is confirmed by the results of our experimental studies and in [33,63]. With the introduction of 10% CS, the drop in strength characteristics is not so significant. The compressive strength decreased by 4.7%, the prism strength decreased by 4.7% and flexural strength decreased by 3.4%. With the introduction of 15–30% CS significant losses in strength characteristics are observed. Thus, the introduction of more than 10% CS instead of a part of the natural coarse aggregate already leads to significant strength losses from 13 to 42%. The drop in the strength characteristics of concrete with the introduction of more than 5% CS is associated with the characteristics of this material. CS has a significantly lower strength than natural coarse aggregate and is also a lighter material. Therefore, the use of CS in the technology of heavy concrete will inevitably lead to a decrease in the strength characteristics [31,32,33,34,35,46,63,64].

Analyzing the obtained dependences of the strength of concrete on the content of coconut shell in the concrete mixture, the following should be noted. The change in the strength is largely due to the rate of water absorption of the coconut shell and the physical and mechanical characteristics of the shell. Coconut shell as a whole has average characteristics that have been determined experimentally and theoretically; at the same time, there is some difference in the characteristics of specific types of nuts and shells. Thus, a factor may arise that should be learned to manage and regulate it. Thus, the conducted experiment is also useful from the point of view of the analysis of the shell itself as a component of concrete. A very important factor in controlling the strength of concrete is to control the characteristics of the shell from the point of view of preventing increased water absorption of the coconut shell, which can lead to an increase in the water demand of the concrete mixture and, in the case of an excessively low water demand for the coconut shell, there may be a risk of surface underwetting and hence problems for the adhesion of the shell to the composite matrix. Thus, this factor is one of the main ones and deserves special attention.

**Figure 13:**
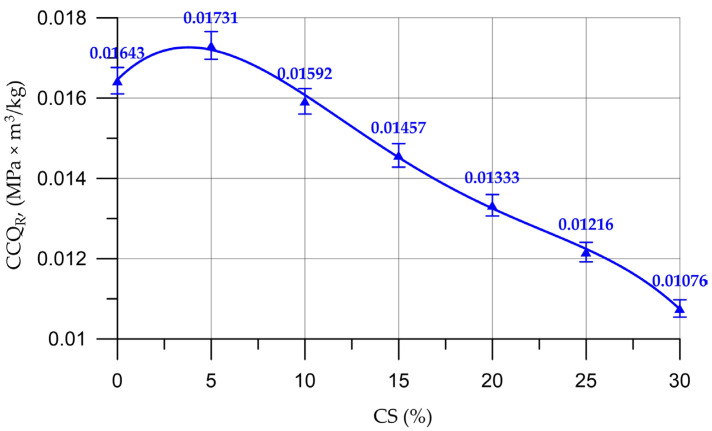
Change in the CCQ of concrete depending on the content of CS.

However, due to its lower density, CS leads to a decrease in the weight of concrete, which contributes to an increase in the CCQ of concrete. The values of the structural quality factors for concrete with different CS contents are shown in Figure 13.

The dependency of the CCQ shown in Figure 13 is approximated by polynomial Function (10) of the following form:

(10) $$CCQ_{R}=0.0164+0.000468\;x\;-7.97\;\times 10^{-5}x^{2}+3.39\times 10^{-6}\;x^{3}-4.885\times 10^{-8}\;x^{4},\;R_{d}^{2}=0.998$$

From Figure 13, it follows that the best value for the coefficient of structural quality is also concrete with a CS content of 5% (up to 6% more than that of the control composition concrete). The *CCQ* values of the control composition concrete and concrete with 10% CS are approximately equal, which confirms the range of effective percentages of CS obtained in the study of the strength characteristics of concrete.

After analyzing the test results, we can say that the use of up to 10% CS instead of a part of natural coarse aggregate is justified and does not lead to significant loss of strength characteristics. The value of the compressive strength when replacing natural coarse aggregate with 5% CS was 38.0 MPa, it was 34.8 MPa at 10% CS, for prismatic strength—28.9 MPa at 5% CS and 26.5 MPa at 10% CS and for bending strength—6.1 MPa at 5% CS and 5.7 MPa at 10% CS.

Above 10% CS, the compressive, flexural and prismatic strengths begin to decrease significantly. When the content of CS is in a percentage from 15% to 30%, the loss of compressive strength ranged from 13% to 40%, the loss of prismatic strength ranged from 13% to 42% and the loss of bending strength ranged from 15% to 41%.

### 3.2. Analysis of the Microstructure of Concrete Samples

**Figure 14:**
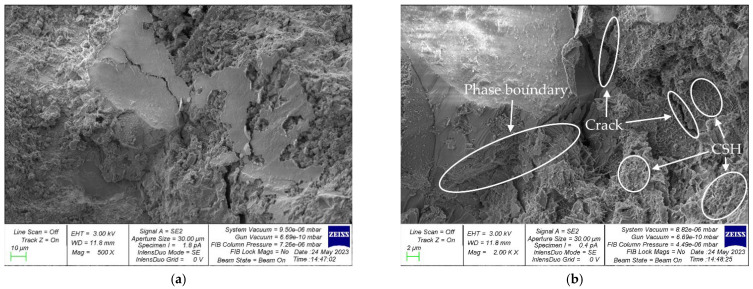
Photographs of the microstructure of the sample illustrating the phase boundary “natural coarse aggregate—cement-sand matrix”: (**a**) 500×; (**b**) 2000×.

**Figure 15:**
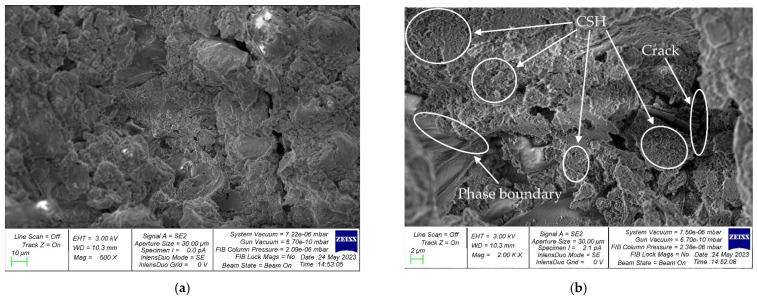
Photographs of the microstructure of the sample illustrating the phase boundary “aggregate of organic origin—cement-sand matrix”: (**a**) 500×; (**b**) 2000×.

Figure 14 and Figure 15 show images of the microstructure of concrete samples with 5% CS content. Figure 14 shows sections of the phase boundary “natural coarse aggregate—cement-sand matrix”, and Figure 15 shows sections of the interface “aggregate of organic origin—cement-sand matrix”.

At the phase boundary “natural coarse aggregate—cement-sand matrix” shown in Figure 14, there are areas of accumulation of CSH and microcracks. A distinctive feature is that the cement–sand matrix envelops the grain of natural coarse aggregate (Figure 14b). In Figure 15, which illustrates the section of the phase boundary “aggregate of organic origin—cement-sand matrix”, one can also see accumulations of calcium hydrosilicates (CSH) and microcracks. Figure 15b clearly illustrates the porous structure of the CS, into which the cement paste penetrates, thereby creating the good adhesion of this aggregate into the cement–sand matrix. Due to the rough and porous surface, CS particles have a high coefficient of adhesion to the cement–sand matrix of concrete, which significantly compacts the material at the interface “aggregate of organic origin—cement-sand matrix”. In addition, due to the absorption of a small amount of water by CS particles during the hardening of the composite, the hydration at the phase boundaries proceeds more intensively. These two factors allow, at certain small percentages of such an organic aggregate, the maintenance of the strength characteristics of concrete at approximately the same level as ordinary concrete by compensating for the lower strength characteristics of coconut shell compared with natural coarse aggregate. The drop in the strength characteristics of concrete with the introduction of more than 5% CS as a replacement for part of the natural coarse aggregate is associated with the characteristics of the CS. CS has a significantly lower strength compared with natural coarse aggregate and is also a lighter material, which, at high shell amounts (more than 10%), leads to a significant decrease in the strength of the concrete [31,32,33,34,35,46,63,64]. This makes it possible to involve almost all cement particles in this process and create additional crystallization centers that increase the adhesion strength of CS particles with the cement–sand matrix of concrete, which is in good agreement with the studies reported in [33,34,63,64].

Summing up the results of experimental studies, it is necessary to discuss the results obtained. As mentioned above, when establishing rational recipe technological factors and identifying the optimal amounts, it is possible to obtain high-quality concrete using CS as an aggregate. This confirms our working hypothesis that was put forward above; the confirmation of this hypothesis is in good agreement with the works of other authors, namely [33,34,63,64]. In this study, the optimal percentage for replacing a part of the coarse aggregate with CS is considered to be an amount of up to 10%. For example, in [33,63], an amount of CS up to 10% was also optimal. This makes it possible to obtain concrete of the required quality without a significant loss of strength. In [34], the authors managed to obtain concrete without a deterioration in performance with 15% of CS introduced instead of part of the coarse aggregate. Additionally, in [64], based on the results of experimental studies, it was established that it is possible to use up to 20% of CS instead of part of the natural coarse aggregate without a significant deterioration in strength characteristics. In our study, the increase in the obtained characteristics was 4.1% for compressive strength, the density decreased to 9.1% and the CCQ increased by 6.1% compared with the control sample. In [19,20,42], the compressive strength decreased to 25% and the density decreased to 7%; the indicator of CCQ also decreased. In [65], the values of the CCQ varies from 0.00989 to 0.01950 for fine-grained concrete depending on the cement type and the amount of microsilica added, which is comparable with the CCQ values in the current study. In [66], the authors managed to increase the CCQ of lightweight concrete from 0.0252 to 0.0331, which exceeds the CCQ values in the current study. The bending strength values in [48], expressed as a percentage of the compressive strength (16.42% and 17.53%), are comparable with the values obtained in the current study (15.7–16.5%). Moreover, the drops in density and compressive strength in the current study (9.1% and 42%, respectively) were greater than in [33] (7.5% and 22%, respectively), which may be due to differences in the proportions of the mixture and the density of the control composition of concrete, as well as in the characteristics of the coconut shell.

It should be noted that at optimal amounts of CS, an important aspect is not only a slight increase in the strength characteristics but also, to some extent, maintaining them at the same level, provided that the density of concrete is simultaneously reduced. This is achieved through the same mechanism that occurs by replacing a heavy and dense aggregate with a light aggregate of plant origin [67]. Reducing the density of the aggregate automatically leads to a reduction in the weight of the concrete, and this is a great advantage for the concrete created on this basis. Concrete with a carefully selected amount of CS (up to 10%) has strength characteristics that are comparable with ordinary concrete and can be used when high requirements for long-term characteristics are not imposed on the concrete [68,69]. It should be noted that we are the first to propose evaluating the feasibility of such replacements using the parameter “coefficient of constructive quality; other researchers have evaluated the effectiveness of introducing CS into concrete in terms of an absolute indicator such as an increase in strength.

In addition to the obvious advantages of the new concrete, namely its environmental friendliness and economy, we would like to emphasize the features of the structure formation of such concrete. We have carried out studies at the micro and macro levels that confirmed the good joint work of the cement–sand matrix and aggregates of plant origin. At the macro level, the effectiveness of such a recipe and such a composition of concrete is confirmed by good strength characteristics. At the micro level, as can be seen in the photographs of the microstructure, the cement–sand matrix–CS grain interface has good adhesion and there is no or little microcracking. This confirms the good joint work and the high degree of adhesion of the aggregate with the cement–sand matrix, which provide good properties for the resulting concrete. It should be noted that the shell is similar to natural crushed stone and has a rough, angular structure that contributes to the creation of a more developed surface and, thereby, good joint work between the aggregate and the cement–sand concrete matrix. Thus, both practically (at the level of test results) and fundamentally (at the level of structure formation processes), with the help of micro- and macro-approaches, the effectiveness of the developed concrete composition is confirmed.

## 4. Conclusions

The formulations of concrete mixtures with different CS contents were developed and tested. The main characteristics, such as density, compressive strength, bending strength and prism strength, as well as the coefficient of constructive quality of concretes, were investigated. The study used normative tests and scanning electron microscopy.

It was established that the density of concrete decreases with an increase in the CS content. The best strength characteristics of concrete were recorded at a CS percentage of 5%, which replaced a part of natural coarse aggregate by volume. The effectiveness of the introduction of CS into concrete was also evaluated in terms of the “coefficient of constructive quality”, which showed the ratio of the strength of the material to its density. Its highest values were recorded when the CS percentage was 5%.

An analysis of the microstructure of concrete containing CS instead of part of the natural coarse aggregate showed that the cement paste penetrated into the pores of the CS, thereby creating good adhesion of this aggregate to the cement–sand matrix. This factor, at small percentages of CS (up to 10%), compensates for the lower strength of CS compared with natural coarse filler; therefore, the strength of the composite does not significantly decrease.

CS contents in concrete of up to 10% are the most rational; a further increase in the content of CS inevitably leads to a significant drop in strength characteristics in comparison with concrete without CS.

The continuation of this research is planned in the direction of studying the complex partial replacement of the binder and aggregates in concrete with vegetable waste and the use of natural fibers as a reinforcing element, as well as the design and manufacture of more environmentally friendly and economical structures from such concretes.

## Figures and Tables

**Table 1:** Physical and mechanical characteristics of Portland cement.

Property	Value
Specific surface area (m^2^/kg)	338
Soundness (mm)	0.5
Fineness, passage through a sieve No 008 (%)	98.1
Setting times (min)	
-start	165
-end	230
Compressive strength (MPa):	
-2 days	18.5
-28 days	49.2

**Table 2:** Mineralogical composition of Portland cement.

Mineral	Content (%)
C_3_S (alite)	66
C_2_S (belite)	14
C_3_A (tricalcium aluminate)	8
C_4_AF (tetracalcium aluminoferrite)	12

**Table 3:** Grain composition and physical characteristics of sand.

Residues on Sieves (%)	Sieve Diameter (mm)						Fineness Modulus
	2.5	1.25	0.63	0.315	0.16	<0.16	
Partial	1.5	2.0	10.5	50.5	34.0	1.5	1.82
Total	1.5	3.5	14.0	64.5	98.5		
Bulk density (kg/m^3^)				1464			
The content of dust and clay particles (%)				0.3			
Content of clay in lumps (%)				0.1			
Organic and contaminant content				No			

**Table 4:** Characteristics of crushed sandstone.

Indicator Title	Actual Value
Particle size (mm)	5–10
Bulk density (kg/m^3^)	1397
Apparent density (kg/m^3^)	2548
Resistance to fragmentation (wt.%)	12.6
The content of lamellar and acicular grains (wt.%)	9.1
Voids (%)	45

**Table 5:** Characteristics of CS filler.

Indicator Title	Actual Value
Particle size (mm)	5–10
Moisture content (%)	4.6
Water absorption (%)	26.1
Bulk density (kg/m^3^)	592
Apparent density (kg/m^3^)	1082

**Table 6:** Concrete Mix Design.

Composition Type	Concrete Mixture Proportion per 1 m^3^							
	Portland Cement (kg/m^3^)	Water (L/m^3^)	Crushed Stone (kg/m^3^)	Coconut Shell (kg/m^3^)	Sand (kg/m^3^)	Poly-Plast-SP1 (%)	Density (kg/m^3^) [49]	Slump (cm) [50]
0 CS	340	195	1002	0	690	0	2227	8.4
5 CS	340	195	952	21	690	0	2198	8.3
10 CS	340	195	901	43	690	0.5	2169	7.9
15 CS	340	195	851	64	690	1.0	2140	7.7
20 CS	340	195	801	85	690	1.0	2111	7.6
25 CS	340	195	751	106	690	1.0	2082	6.8
30 CS	340	195	701	128	690	1.5	2053	6.7

**Table 7:** Change in the characteristics of concrete (∆) in %, depending on the amount of CS replacing natural coarse aggregate in %.

Characteristics of Concrete	∆ in % with Coarse Aggregate from Coconut Shell; % by Volume of Coarse Aggregate						
	0	5	10	15	20	25	30
Density (kg/m^3^)	0	−2.0	−3.0	−4.2	−5.5	−7.4	−9.1
Compressive strength (MPa)	0	4.1	−4.7	−12.6	−25.2	−34.2	−39.5
Prism strength (MPa)	0	4.0	−4.7	−13.3	−23.7	−34.9	−41.4
Bending strength (MPa)	0	3.4	−3.4	−15.3	−23.7	−33.9	−40.7

## Data Availability

The study did not report any data.

## References

[1] Khan S.S., Ali M. (2022). Short-Term and Long-Term Needs for Sustainable Concrete—An Overview. Eng. Proc..

[2] Jang H.-J., Ahn Y.-H., Tae S.-H. (2022). Proposal of Major Environmental Impact Categories of Construction Materials Based on Life Cycle Impact Assessments. Materials.

[3] WMO (2019). WMO Greenhouse Gas Bulletin—The State of the Greenhouse Gases in the Atmosphere Based on Global Observations through 2018.

[4] Katman H.Y.B., Khai W.J., Bheel N., Kırgız M.S., Kumar A., Khatib J., Benjeddou O. (2022). Workability, Strength, Modulus of Elasticity, and Permeability Feature of Wheat Straw Ash-Incorporated Hydraulic Cement Concrete. Buildings.

[5] Ramasubramani R., Gunasekaran K. (2021). Sustainable Alternate Materials for Concrete Production from Renewable Source and Waste. Sustainability.

[6] Shcherban’ E.M., Stel’makh S.A., Beskopylny A.N., Mailyan L.R., Meskhi B., Shilov A.A., Chernil’nik A., Özkılıç Y.O., Aksoylu C. (2022). Normal-Weight Concrete with Improved Stress–Strain Characteristics Reinforced with Dispersed Coconut Fibers. Appl. Sci..

[7] Beskopylny A.N., Stel’makh S.A., Shcherban’ E.M., Mailyan L.R., Meskhi B., Smolyanichenko A.S., Varavka V., Beskopylny N., Dotsenko N. (2022). Influence of Electromagnetic Activation of Cement Paste and Nano-Modification by Rice Straw Biochar on the Structure and Characteristics of Concrete. J. Compos. Sci..

[8] Beskopylny A.N., Stel’makh S.A., Shcherban E.M., Mailyan L.R., Meskhi B., Shilov A.A., Beskopylny N., Chernil’nik A. (2022). Enhanced Performance of Concrete Dispersedly Reinforced with Sisal Fibers. Appl. Sci..

[9] Shcherban’ E.M., Stel’makh S.A., Beskopylny A.N., Mailyan L.R., Meskhi B., Varavka V., Beskopylny N., El’shaeva D. (2022). Enhanced Eco-Friendly Concrete Nano-Change with Eggshell Powder. Appl. Sci..

[10] Olanipekun E.A., Olusola K.O., Ata O. (2006). A comparative study of concrete properties using coconut shell and palm kernel shell as coarse aggregates. Build. Environ..

[11] Beskopylny A.N., Shcherban’ E.M., Stel’makh S.A., Mailyan L.R., Meskhi B., Evtushenko A., El’shaeva D., Chernil’nik A. (2023). Improving the Physical and Mechanical Characteristics of Modified Aerated Concrete by Reinforcing with Plant Fibers. Fibers.

[12] Gunasekaran K., Annadurai R., Kumar P.S. (2015). A study on some durability properties of coconut shell aggregate concrete. Mater. Struct..

[13] Tomar R., Kishore K., Parihar H.S., Gupta N. (2021). A comprehensive study of waste coconut shell aggregate as raw material in concrete. Mater. Today Proc..

[14] Beskopylny A.N., Stel’makh S.A., Shcherban’ E.M., Mailyan L.R., Meskhi B., Shilov A.A., Chernil’nik A., El’shaeva D. (2023). Effect of Walnut-Shell Additive on the Structure and Characteristics of Concrete. Materials.

[15] Beskopylny A.N., Shcherban’ E.M., Stel’makh S.A., Meskhi B., Shilov A.A., Varavka V., Evtushenko A., Özkılıç Y.O., Aksoylu C., Karalar M. (2022). Composition Component Influence on Concrete Properties with the Additive of Rubber Tree Seed Shells. Appl. Sci..

[16] Coffetti D., Crotti E., Gazzaniga G., Carrara M., Pastore T., Coppola L. (2022). Pathways towards sustainable concrete. Cem. Concr. Res..

[17] Khan K., Salami B.A., Jamal A., Amin M.N., Usman M., Al-Faiad M.A., Abu-Arab A.M., Iqbal M. (2022). Prediction Models for Estimating Compressive Strength of Concrete Made of Manufactured Sand Using Gene Expression Programming Model. Materials.

[18] Almadani M., Razak R.A., Abdullah M.M.A.B., Mohamed R. (2022). Geopolymer-Based Artificial Aggregates: A Review on Methods of Producing, Properties, and Improving Techniques. Materials.

[19] Gerges N.N., Issa C.A., Sleiman E., Aintrazi S., Saadeddine J., Abboud R., Antoun M. (2022). Eco-Friendly Optimum Structural Concrete Mix Design. Sustainability.

[20] Liu H., Li Q., Quan H., Xu X., Wang Q., Ni S. (2022). Assessment on the Properties of Biomass-Aggregate Geopolymer Concrete. Appl. Sci..

[21] Marey H., Kozma G., Szabó G. (2022). Effects of Using Green Concrete Materials on the CO_2_ Emissions of the Residential Building Sector in Egypt. Sustainability.

[22] Liu H., Li Q., Ni S. (2022). Assessment of the engineering properties of biomass recycled aggregate concrete developed from coconut shells. Constr. Build. Mater..

[23] Nunes L.A., Silva M.L.S., Gerber J.Z., Kalid R.A. (2020). Waste green coconut shells: Diagnosis of the disposal and applications for use in other products. J. Clean. Prod..

[24] Srivani G., Vamsi Mohan U. (2023). Study on strength properties of concrete by partial replacement of cement with sugarcane bagasse ash and coarse aggregate with coconut shells. Mater. Today Proc..

[25] Bari H., Salam M.A., Safiuddin M. (2021). Fresh and hardened properties of brick aggregate concrete including coconut shell as a partial replacement of coarse aggregate. Constr. Build. Mater..

[26] Prithika A.J., Sekar S.K. (2016). Mechanical and fracture characteristics of Eco-friendly concrete produced using coconut shell, ground granulated blast furnace slag and manufactured sand. Constr. Build. Mater..

[27] Janani S., Kulanthaivel P., Sowndarya G., Srivishnu H., Shanjayvel P.G. (2022). Study of coconut shell as coarse aggregate in light weight concrete- a review. Mater. Today Proc..

[28] Prakash R., Divyah N., Srividhya S., Avudaiappan S., Amran M., Naidu Raman S., Guindos P., Vatin N.I., Fediuk R. (2022). Effect of Steel Fiber on the Strength and Flexural Characteristics of Coconut Shell Concrete Partially Blended with Fly Ash. Materials.

[29] Mhaya A.M., Shahidan S., Algaifi H.A., Zuki S.S.M., Benjeddou O., Ibrahim M.H.W., Huseien G.F. (2022). Thermal Conductivity of Coconut Shell-Incorporated Concrete: A Systematic Assessment via Theory and Experiment. Sustainability.

[30] Mhaya A.M., Algaifi H.A., Shahidan S., Zuki S.S.M., Azmi M.A.M., Ibrahim M.H.W., Huseien G.F. (2022). Systematic Evaluation of Permeability of Concrete Incorporating Coconut Shell as Replacement of Fine Aggregate. Materials.

[31] Raja K.C.P., Thaniarasu I., Elkotb M.A., Ansari K., Saleel C.A. (2021). Shrinkage Study and Strength Aspects of Concrete with Foundry Sand and Coconut Shell as a Partial Replacement for Coarse and Fine Aggregate. Materials.

[32] Sekar A., Kandasamy G. (2019). Study on Durability Properties of Coconut Shell Concrete with Coconut Fiber. Buildings.

[33] Kanojia A., Jain S.K. (2017). Performance of coconut shell as coarse aggregate in concrete. Constr. Build. Mater..

[34] Bhoj S., Manoj A., Bhaskar S. (2023). Usage potential and benefits of processed coconut shells in concrete as coarse aggregates. Mater. Today Proc..

[35] Itam Z., Johar A.D., Syamsir A., Zainoodin M., Shaikh Ahmad Fadzil S.M.M., Beddu S. (2022). Utilization of coconut shell as a supplementary cementitious material in concrete. Mater. Today Proc..

[36] Bari H., Safiuddin M., Salam M.A. (2021). Microstructure of Structural Lightweight Concrete Incorporating Coconut Shell as a Partial Replacement of Brick Aggregate and Its Influence on Compressive Strength. Sustainability.

[37] Sujatha A., Deepa Balakrishnan S. (2023). Properties of high strength lightweight concrete incorporating crushed coconut shells as coarse aggregate. Mater. Today Proc..

[38] Palanisamy M., Kolandasamy P., Awoyera P., Gobinath R., Muthusamy S., Krishnasamy T.R., Viloria A. (2020). Permeability properties of lightweight self-consolidating concrete made with coconut shell aggregate. J. Mater. Res. Technol..

[39] Aziz W., Aslam M., Ejaz M.F., Ali M.J., Ahmad R., Wajeeh-ul-Hassan Raza M., Khan A. (2022). Mechanical properties, drying shrinkage and structural performance of coconut shell lightweight concrete. Structures.

[40] Krishnaswami N., Velusamy S., Palanisamy C., Prakash G., Loganathan K.K., Moorthy J. (2022). Experimental studies on light weight concrete using gib & coconut shell in concrete. Mater. Today Proc..

[41] Soumya S., Pennarasi G., Gunasekaran K. (2019). Study on the reinforced manhole cover slab using coconut shell aggregate concrete. Mater. Today Proc..

[42] Herring T.C., Nyomboi T., Thuo J.N. (2022). Ductility and cracking behavior of reinforced coconut shell concrete beams incorporated with coconut shell ash. Results Eng..

[43] Gunasekaran K., Annadurai R., Kumar P.S. (2013). Plastic shrinkage and deflection characteristics of coconut shell concrete slab. Constr. Build. Mater..

[44] Sekar A., Kandasamy G. (2018). Optimization of Coconut Fiber in Coconut Shell Concrete and Its Mechanical and Bond Properties. Materials.

[45] Thangasamy L., Kandasamy G. (2020). Behavior of Steel–Coconut Shell Concrete–Steel Composite Beam without and with Shear Studs under Flexural Load. Materials.

[46] Gunasekaran K., Annadurai R., Kumar P.S. (2013). Study on reinforced lightweight coconut shell concrete beam behavior under shear. Mater. Des..

[47] Gunasekaran K., Annadurai R., Chandar S.P., Anandh S. (2017). Study for the relevance of coconut shell aggregate concrete non-pressure pipe. Ain Shams Eng. J..

[48] Gunasekaran K., Kumar P.S., Lakshmipathy M. (2011). Mechanical and bond properties of coconut shell concrete. Constr. Build. Mater..

[49] (2019). Testing Fresh Concrete. Part 6. Density.

[50] (2019). Testing Fresh Concrete. Part 2. Slump Test.

[51] (2017). Concrete. General Specifications..

[52] Thilagashanthi T., Gunasekaran K., Satyanarayanan K.S. (2021). Microstructural pore analysis using SEM and ImageJ on the absorption of treated coconut shell aggregate. J. Clean. Prod..

[53] (2018). Concretes. Methods for Strength Determination Using Reference Specimens.

[54] (2021). Testing Hardened Concrete—Part 1: Shape, Dimensions and other Requirements of Specimens and Moulds.

[55] (2021). Testing Hardened Concrete—Part 2: Making and Curing Specimens for Strength Tests.

[56] (2021). Testing Hardened Concrete—Part 3: Compressive Strength of Test Specimens.

[57] (2021). Testing Hardened Concrete—Part 4: Compressive Strength—Specification for Testing Machines.

[58] (2021). Testing Hardened Concrete—Part 5: Flexural Strength of Test Specimens.

[59] (2005). Concretes. Methods of Prismatic, Compressive Strength, Modulus of Elasticity and Poisson’s Ratio Determination.

[60] (2021). Concretes. Methods of Determination of Density.

[61] Stel’makh S.A., Shcherban’ E.M., Beskopylny A.N., Mailyan L.R., Meskhi B., Beskopylny N., Dotsenko N., Kotenko M. (2022). Influence of Recipe Factors on the Structure and Properties of Non-Autoclaved Aerated Concrete of Increased Strength. Appl. Sci..

[62] Shcherban’ E.M., Stel’makh S.A., Beskopylny A., Mailyan L.R., Meskhi B., Varavka V. (2021). Nanomodification of Lightweight Fiber Reinforced Concrete with Micro Silica and Its Influence on the Constructive Quality Coefficient. Materials.

[63] Prakash R., Thenmozhi R., Raman S.N., Subramanian C., Divyah N. (2021). An investigation of key mechanical and durability properties of coconut shell concrete with partial replacement of fly ash. Struct. Concr..

[64] Tangadagi R.B., Manjunatha M., Preethi S., Bharath A., Reshma T.V. (2021). Strength characteristics of concrete using coconut shell as a coarse aggregate—A sustainable approach. Mater. Today Proc..

[65] Baranova A.A., Yazina O.I., Bobrova A.A., Rudykh K.N. (2018). Effect of quantity of silica fume on the coefficients of constructive quality fine-grained concrete and structural foam concrete. Mod. Technol. Sci. Technol. Prog..

[66] Rylova T., Lakhtaryna S., Yegorova O. (2018). Lightweight structural concrete with an increased coefficient of structural quality. Proc. DonNACEA.

[67] Gunasekaran K., Annadurai R., Kumar P.S. (2013). Study on reinforced lightweight coconut shell concrete beam behavior under flexure. Mater. Des..

[68] Liu H., Li Q., Wang P. (2023). Assessment of the engineering properties and economic advantage of recycled aggregate concrete developed from waste clay bricks and coconut shells. J. Build. Eng..

[69] Ni S., Liu H., Li Q., Quan H., Gheibi M., Fathollahi-Fard A.M., Tian G. (2022). Assessment of the engineering properties, carbon dioxide emission and economic of biomass recycled aggregate concrete: A novel approach for building green concretes. J. Clean. Prod..

